# Monitoring in vivo behavior of size-dependent fluorescent particles as a model fine dust

**DOI:** 10.1186/s12951-022-01419-4

**Published:** 2022-05-12

**Authors:** Taewoong Son, Youn-Joo Cho, Hyunseung Lee, Mi Young Cho, Byeongwoo Goh, Hyun Min Kim, Phan Thi Ngoc Hoa, Sun-Hee Cho, Young-Jun Park, Hye Sun Park, Kwan Soo Hong

**Affiliations:** 1 Research Center for Bioconvergence Analysis, Korea Basic Science Institute, Cheongju, 28119 South Korea; 2 Environmental Disease Research Center, Korea Research Institute of Bioscience and Biotechnology, Daejeon, 34141 South Korea; 3 Graduate School of Analytical Science and Technology, Chungnam National University, Daejeon, 34134 South Korea; 4 Present Address: SKKU Advanced Institute of Nanotechnology (SAINT), School of Chemical Engineering, Sungkyunkwan University, Suwon, 16419 South Korea

**Keywords:** Air pollution, Particulate matter, Silica particle, Biodistribution, In vivo image-tracking

## Abstract

**Background:**

There has been growing concern regarding the impact of air pollution, especially fine dust, on human health. However, it is difficult to estimate the toxicity of fine dust on the human body because of its diverse effects depending on the composition and environmental factors.

**Results:**

In this study, we focused on the difference in the biodistribution of fine dust according to the size distribution of particulate matter after inhalation into the body to predict its impact on human health. We synthesized Cy7-doped silica particulate matters (CSPMs) having different particle sizes and employed them as model fine dust, and studied their whole-body in vivo biodistribution in BALB/c nude mice. Image-tracking and quantitative and qualitative analyses were performed on the ex vivo organs and tissues. Additionally, flow cytometric analysis of single cells isolated from the lungs was performed. Smaller particles with a diameter of less than 100 nm (CSPM0.1) were observed to be removed relatively rapidly from the lungs upon initial inhalation. However, they were confirmed to accumulate continuously over 4 weeks of observation. In particular, smaller particles were found to spread rapidly to other organs during the early stages of inhalation.

**Conclusions:**

The results show in vivo behavioral differences that arisen from particle size through mouse experimental model. Although these are far from the human inhalation studies, it provides information that can help predict the effect of fine dust on human health. This study might provide with insights on association between CSPM0.1 accumulation in several organs including the lungs and adverse effect to underlying diseases in the organs.

**Graphical Abstract:**

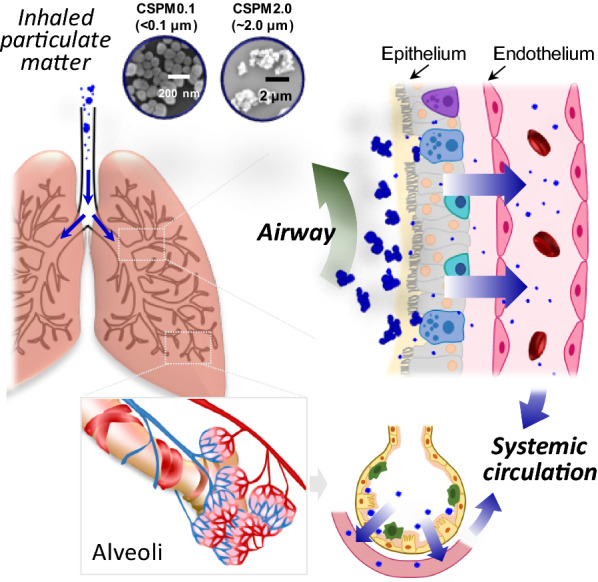

**Supplementary Information:**

The online version contains supplementary material available at 10.1186/s12951-022-01419-4.

## Background

Air pollution is considered to be one of the most serious environmental problems worldwide because of its impact on human health [1, 2]. Particulate matter (PM), a major contributor to air pollution, is a heterogeneous mixture of solid and liquid elements in the air [3, 4]. Inhalation of PM into the body can cause severe respiratory diseases, such as asthma, pneumonia, and even lung cancer, and can also increase the severity of existing diseases [5, 6]. Social interest in the effects of PM is expanding to various research fields, such as identifying and predicting occurrence phenomena, developing detection devices and emission reduction technologies, and evaluating the health effects of PM to preserve human life.

The effect of PM on the human body is compounded by the size or morphology and the degree of toxicity of the constituents [7–9]. Physical characteristics, such as particle size, shape, surface charge, or surface area, are factors that directly affect the biodistribution of particles upon injection in vivo. They are related to in vivo circulation time and tissue permeability. In particular, it has been reported that the smaller the particle size, the higher the cellular uptake, and the more positive the particle surface charge, the greater the transport efficiency of the paracellular pathway in particle internalization [10]. The constituents of PM are distributed in various ways depending on the cause of occurrence and secondary pollution according to the air flow, and it is difficult to generalize the effects of PM toxicity on the human body due to variations among countries and regions in various environments [11]. However, these toxicity issues are strongly influenced by the size of PM [12]. A generalization of the impact of PM size on human health could significantly facilitate the prediction of its toxicity.

It is not easy to directly predict the toxicity to the human body from the distribution pattern of particles. It is known that fine dusts containing chemical components cause deleterious effects such as inflammatory processes by redox reaction in mitochondria or generation of reactive oxygen species after cellular internalization [13]. It will immediately begin to affect the cells and tissues that it comes in contact with. Importantly, inhaled particles deposited in the respiratory tract can travel into the bloodstream and then to other organs of the body, depending on the particle size, chemical composition, surface charge and agglomeration. Particles translocated to other organs can selectively accumulate contaminants in certain tissues of the body or interact with other substances, increasing toxicity [14]. For example, it has been reported that hydrophobic organic compounds from the fine dust accumulate in lipid tissues and cause fatty liver disease [15]. As such, the biodistribution and residence time of the particles can be a clue as to how toxic the particles can be to the tissues or cells in which they reside.

PM exhibits different deposition and distribution patterns in different respiratory tracts depending on its size [16]. Understanding the biological distribution of PM can enable prediction of its effects on human organs, as the toxic effects on surrounding organs depend on the pathway through which it travels and the duration of residence and accumulation after inhalation.

Based on the general criteria of aerodynamic diameters, PM is classified into coarse particles with a diameter of less than 10 μm (PM_10_), fine particles with a diameter of less than 2.5 μm (PM_2.5_), and ultra-fine particles with a diameter of less than 0.1 μm (PM_0.1_) [7, 9]. Larger (PM_10_) particles are primarily deposited by inertial impaction in large airway regions, including the oropharynx, trachea, and bronchi. The deposition of smaller particles in the trachea and bronchi occurs primarily through fast inhalation [17, 18]. PM_2.5_ particles are deposited via gravitational sedimentation along smaller airways, and particles smaller than 0.5 μm in diameter reach the alveoli by Brownian diffusion and are deposited there [19, 20].

Recent studies have shown that ultrafine particles have a greater mass-based toxic effect than larger particles because they have a greater number of particles and a larger surface area than smaller particles of the same mass. There is an urgent need for research on the effects of small particles [21]. Although information on the biodistribution and assessment of the health effects of fine particles is available in several reports, only in vivo or ex vivo imaging using radioisotope-labeled or optical fluorescent-labeled materials as PMs has been attempted in a fragmented manner [18, 22]. Biodistribution analysis of fine or ultra-fine particles requires a comprehensive analysis including in vivo tracking along with in vitro tissue analysis to investigate particle interactions at the cellular level. In particular, to better understand the effects of potentially toxic PMs on biological samples, it is necessary to characterize and predict their interactions and behaviors in living subjects. Research reports on human inhalation of PMs are difficult to generalize because they are very complex and various variables affect them. Therefore, there are limitations in extending the results from mouse model experiments to human implications. Regardless, it is expected that the results occurring at the cellular level after reaching lung cells will be similar. Although there are differences that arise from the size of the organism, such as airway diameter and alveolar size, mouse lungs are anatomically similar to human lungs. Similar to the human organs, the mouse lung is subdivided into lobes of lung parenchyma containing a branching bronchial tree and is vascularized by the pulmonary circulation [23, 24]. Therefore, an experiment was conducted to understand the in vivo particle behavior in mice that can provide valuable information about the behavior of fine dust in the human body.

**Fig. 1:**
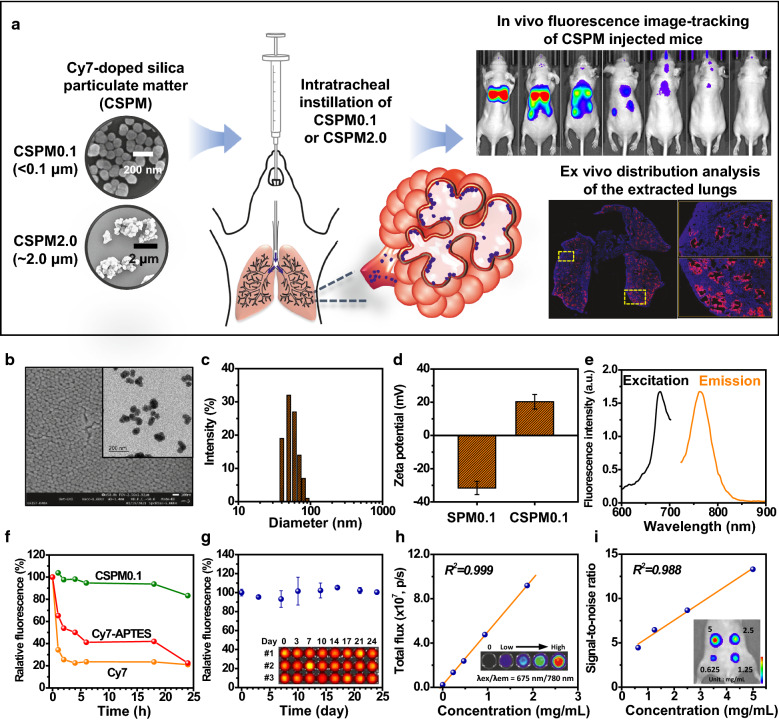
Synthesis and characterization of CSPM. **a** Schematic illustration of the synthesis of CSPMs with different particle sizes and their in vivo biodistribution imaging studies. **b** A representative FE-SEM image (scale bar = 100 nm) and HR-TEM image (the inset, scale bar = 200 nm) of the synthesized CSPM0.1. **c** Size distribution analysis of the synthesized CSPM0.1. **d** Surface zeta potentials of silica particulate matter (SPM) without dye-doping and CSPM0.1. **e** Fluorescence spectra (excitation and emission scan modes) of CSPM0.1. **f** Fluorescence intensity profiles of CSPM0.1, Cy7-APTES, and Cy7 with time. **g** In vitro dye release profiles of CSPM0.1. Fluorescence intensity linearity in **h** in vitro and **i** in vivo environments for CSPM0.1. CSPM0.1 was subcutaneously injected into the mouse abdomen at different concentrations

In this study, silica-based particles, which are highly amenable to engineering and modification, were used as model materials to simulate particulate matter, and near-infrared (NIR) dyes suitable for bioimaging were doped inside them to study their biodistribution in mice. In particular, small particles with a diameter of less than 100 nm and particles with a diameter of 2 μm or more were separately synthesized and employed for the comparative analysis of distribution patterns. In addition, quantitative analysis of tissue images was performed and cellular uptake information was observed over an extended period (Fig. 1a).

## Results and discussion

### Synthesis and characterization of silica-based particulate matters

As model materials for PM, silica-based particles were employed and synthesized with a controlled size distribution using a modified Stöber method [25–28]. Incorporation of cyanine dyes into the silica particles facilitates optical imaging with deeper in vivo penetration in the NIR region. The synthesized CSPM0.1 had a uniform diameter of 54.0 ± 0.9 nm with a popcorn-like shape (Fig. 1b, c). The surface zeta potential values shifted from − 31.5 ± 4.7 to 20.5 ± 3.9 mV due to the presence of the exposed amine groups on the particle surface after Cy7-APTES was doped into the silica matrix (Fig. 1d). The amount of Cy7 encapsulation in the particles was optimized in consideration of the quenching effect of Cy7, and the number of Cy7 molecules encapsulated per mg of particles was quantified as 5.85 nmol/mg (Additional file 1: Fig. S1). The main peaks in the excitation/emission fluorescence spectra of CSPM0.1 (Fig. 1e) appeared at 675/780 nm. Evaluation of the fluorescence stability of CSPM0.1 (Fig. 1f) showed that CSPM0.1 maintained its fluorescence properties compared to Cy7 and Cy7-APTES in its free form. This implies that the release of a fraction of the Cy7 dye from the particles is expected to have only a negligible effect on the fluorescence signal. In addition, relatively stable fluorescence signals were observed for more than 3 weeks, with negligible variations due to the release of the dye (Fig. 1g).

In addition, upon monitoring the particles incubated in saline and 10% FBS containing medium for an extended period (Additional file 1: Fig. S2a), it was confirmed that the morphological stability of the particles was maintained without agglomeration. We also investigated the stability of particle fluorescence to reactive oxygen species (ROS), which may occur after particle injection in vivo and has been reported to reduce the stability of fluorescent dyes [29, 30]. As a result of analyzing the fluorescence signals for CSPM and the Cy7 dyes in various ROS environments, it was confirmed that the fluorescence signals of CSPMs were stably maintained in the ROS environments compared to the results of the Cy7 dyes (Additional file 1: Fig. S3).

To assess the fluorescence imaging performance of CSPM0.1, the signal for each concentration was measured in vitro and in vivo using an in vivo imaging system, and it was confirmed that the fluorescence signal was proportionate to the concentration (Fig. 1h, i).

### Fluorescence imaging studies of CSPM injected mice

**Fig. 2:**
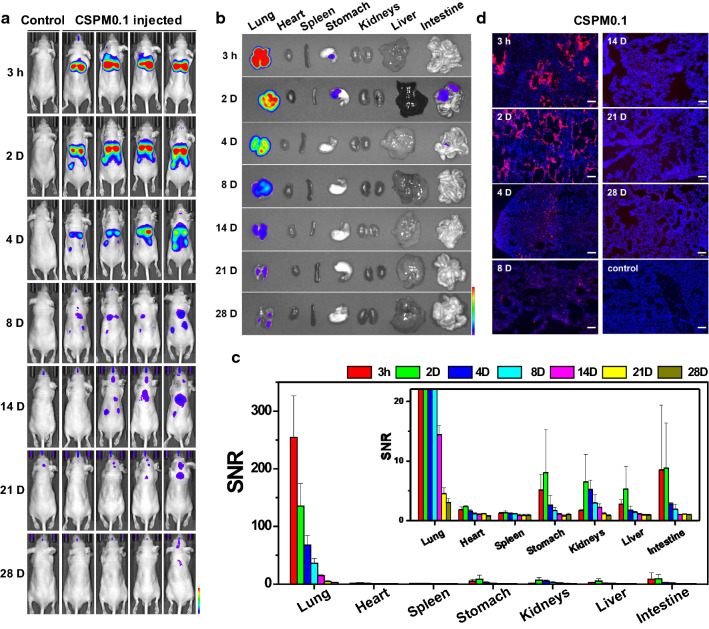
In vivo fluorescence imaging and biodistribution study of CSPM0.1. **a** Representative real-time in vivo fluorescence images of CSPM0.1 injected intratracheally into the mice. **b** Ex vivo fluorescence images of the major organs from the injected mice. **c** SNR obtained from the fluorescence intensities of the major organs at different time-points. **d** Fluorescence microscopy images of the dissected lung tissues. Red and blue colors indicate CSPMs and DAPI staining, respectively. The data shown are averages of > 3 independent experiments (scale bar = 100 µm)

To investigate the biodistribution of the ultra-fine particles entering the body via inhalation, the synthesized CSPM0.1 were injected intratracheally into the mice. In vivo whole-body fluorescence images were obtained at different time-points after injection, and the major organs were extracted and imaged ex vivo from the sacrificed mice, as shown in Fig. 2a, b. During in vivo imaging analysis, a decrease in the penetration depth due to interference from ribs or other organs inevitably leads to a decrease in the fluorescence signal, which can be quantitatively complemented by ex vivo organ imaging. Signals detected in the digestive tract appear to have been swallowed during instillation. Figure 2c shows the signal-to-noise ratios (SNRs) obtained for the fluorescence intensities of the excised organs. Compared to the initial value, the SNR in the lungs decreased to approximately 53.1% after 2 days of injection, 5.7% after 2 weeks, and 1.2% after 4 weeks, indicating that the signal was detectable even after 4 weeks of injection. As shown in the inset of Fig. 2c, the signals from the kidneys, liver, and intestine were significantly weaker than those from the lungs. However, they were not negligible. The residual signal fractions in the kidneys, liver, and intestine 2 days after injection were measured to be 2.6, 2.1, and 3.4%, respectively, which were higher than the 1.8% measured in the lungs after 3 weeks. Additional file 1: Fig. S6 shows the results of ICP-OES quantitative analysis of Si contained in the excised organs. Compared to the initial value, the quantitative Si in the lungs decreased to approximately 4.5% after 2 weeks of injection and 2.9% after 4 weeks. This shows a similar trend to the SNR results. The similarity between the signal and quantitative indicates that the particle quantification is possible even with fluorescence imaging. This provides important information on the initial flow of the particles injected through the respiratory tract from the pulmonary to systemic circulation. The distribution of the particles in the excised lung tissue over time is illustrated in Fig. 2d. In the early stages after injection, a significant amount of particles was found to be deposited around the bronchioles and alveoli in the lung tissue along the main bronchial inhalation route. The large amount of agglomerated particles that was found to be deposited in the initial stages after injection was not observed after 4 weeks; however, small particles penetrated the tissue and remained scattered between the cells, indicating relatively long time effect of CSPM0.1 accumulation in deep lung tissue after 4 weeks to lung function. The intensity of the fluorescence signals from the particles declined with the passage of time. However, small signals from the sporadically scattered particles were observed after 4 weeks.

**Fig. 3:**
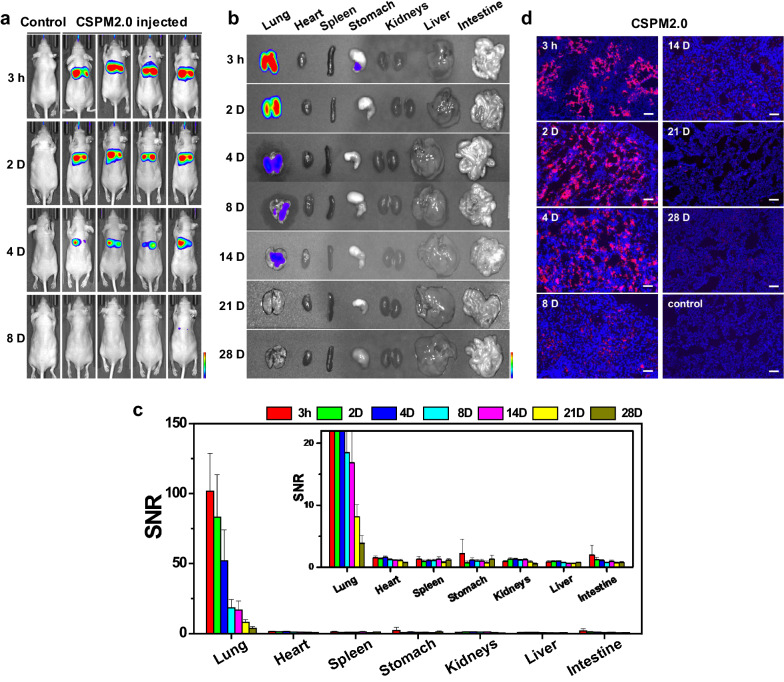
In vivo fluorescence imaging and biodistribution study of CSPM2.0. **a** Representative real-time in vivo fluorescence images of CSPM2.0 injected intratracheally into the mice. **b** Ex vivo fluorescence images of the major organs from the injected mice. **c** SNR obtained from the fluorescence intensities of the major organs at different time-points. **d** Fluorescence microscopy images of the dissected lung tissues. Red and blue colors indicate CSPMs and DAPI staining, respectively. The data shown are averages of > 3 independent experiments (scale bar = 100 µm)

The synthesized CSPM2.0 is designed to have similar properties compared to CSPM0.1 but with a relatively large particle size (Additional file 1: Figs. S2, S4, S5). CSPM2.0 has a diameter of approximately 2 μm with non-uniform agglomeration of small particles, indicating that it is similar to actual fine PM [31]. Figure 3 depicts the results of the in vivo fluorescence imaging and biodistribution studies performed on the CSPM2.0 injected mice. CSPM2.0 was injected intratracheally into the mice at the same concentration as CSPM0.1. In the in vitro fluorescence signal analysis of CSPM2.0, residual signals were detected at 81.8 and 16.6% at 2 days and 2 weeks after injection, respectively, which were 1.5 and 2.9 times higher than those of CSPM0.1. In addition, the fluorescence intensity declined at a slow rate in the initial stages (Fig. 3a–c). In contrast, the organs other than the lungs showed significantly higher penetration of CSPM0.1 than CSPM2.0, indicating that smaller particles such as CSPM0.1 may be partly associated to adverse effect to underlying diseases in the organs of stomach, kidneys, liver, and intestine. From the distribution of the particles in the lung tissues (Fig. 3d), it was found that the particles deposited in the bronchi and alveoli at the beginning of the infusion persisted for up to 4 days, after which a significant amount of particles disappeared from the initial deposition area at a rapid rate, and only some of them penetrated the lung tissue. Although there was significant deposition in the lung tissue in the initial stages, the particles did not reach the systemic circulation due to their rapid removal from the lung tissue and low permeation into the tissue. In fact, since this study examines the in vivo distribution of model fine dust particles, silica-based PM with low toxicity was used (Additional file 1: Fig. S7) [32]. However, the residence time of the particles in the body is significant for toxic materials that are actually present in polluted environments.

### Flow cytometric and qualitative analysis of the excised lungs

**Fig. 4:**
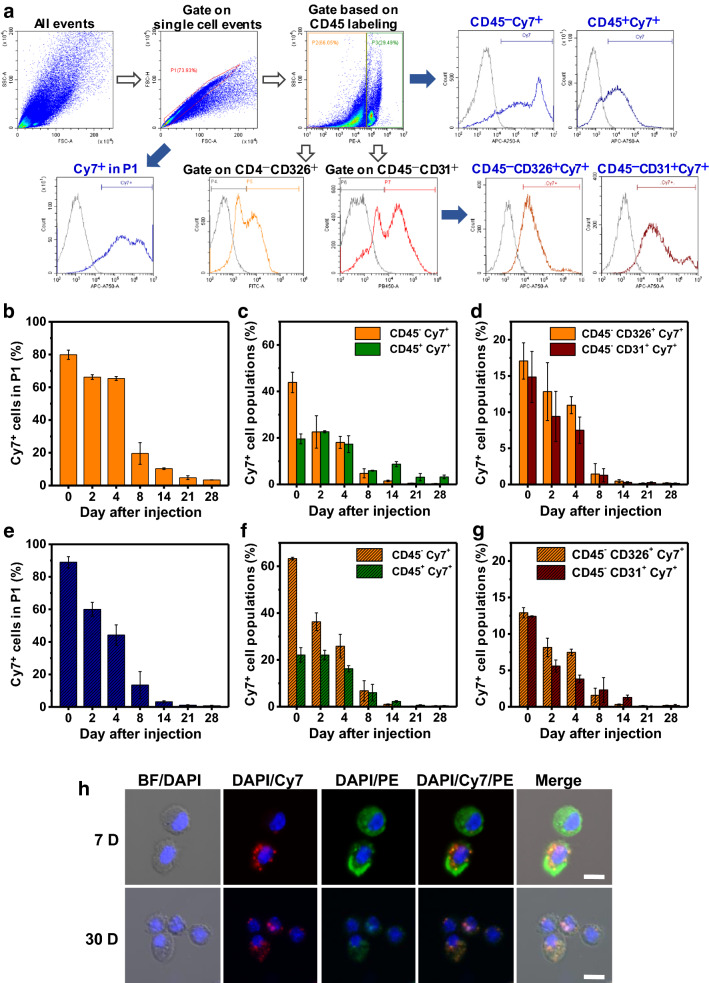
Flow cytometric and microscopic analysis of single-cells obtained from the excised lungs. **a** Gating strategy to identify epithelial and endothelial cells and leukocytes. **b** Qualitative analysis of cellular uptake of CSPM0.1 in lung cells. Populations of CSPM0.1-labeled **c** stromal cells (CD45^−^) and leukocytes (CD45^+^) and **d** epithelial (CD45^−^CD326^+^) and endothelial cells (CD45^−^CD31^+^). **e** Qualitative analysis of cellular uptake of CSPM2.0 in lung cells. Populations of CSPM2.0 labeled **f** stromal cells (CD45^−^) and leukocytes (CD45^+^) and **g** epithelial (CD45^−^CD326^+^) and endothelial cells (CD45^−^CD31^+^). **h** Cellular microscopy images of CSPM-labeled cells. Red, green, and blue colors represent CSPMs, CD45 antibodies, and DAPI staining, respectively (scale bar = 10 µm)

Fine particles inhaled into the lungs can cause diseases, such as allergic asthma due to direct or indirect interactions with the various types of cells that constitute the lungs [33]. Interactions between PM and cells can occur through a variety of pathways, including simple physical accumulation on the tissue surfaces, migration through cell–cell junctions, and cellular internalization with secondary effects on cellular functions [10]. Since the sole analysis of ex vivo image signals can yield limited information, it is necessary to analyze each cell unit according to the types of lung cells. In this study, individual cells in the lung tissues were classified according to their characteristics, and qualitative analysis of the cellular internalization of the CSPMs was performed (Fig. 4a and Additional file 1: Table S1). The leukocyte common antigen, CD45, has been used to classify leukocytes (CD45^+^) and stromal cells (CD45^−^), which is expressed in all hematopoietic cells, including lymphocytes, monocytes, macrophages and fibroblasts. EpCAM (Epithelial Cell Adhesion Molecule, CD326) and PECAM-1 (Platelet Endothelial Cell Adhesion Molecule-1, CD31) were used to classify stromal cells (CD45^−^) into epithelial cells (CD45^−^CD326^+^) and endothelial cells (CD45^−^CD31^+^) [34]. Figure 4b and e depict the Cy7-positive cells for single cells obtained from different parts of the entire lung tissue after the injection of CSPM0.1 and CSPM2.0, respectively. A larger decrease in the fraction of Cy7-positive cells with time after injection was observed in the case of CSPM2.0 injection. Combining these results with those obtained through ex vivo imaging, in the case of CSPM2.0 injection, it can be concluded that the strong signal obtained upon ex vivo imaging at the initial stages after injection accounts for a significant portion of the state in which the particles are physically trapped between the tissues. This is attributed to the weak attachment of the particles with the cells, which results in their easy removal upon the isolation of single cells. The proportion of Cy7-positive cells in the total single cells showed a clear difference in the population between CSPM0.1 and CSPM2.0 at 2 and 4 weeks after injection. For CSPM0.1, the populations after 2 and 4 weeks were 10.3% and 3.4%, respectively, whereas for CSPM2.0, the corresponding populations were 3.2% and 0.8%. After 4 weeks of inhalation of the fine PM, the retention of CSPM0.1 in the lung cells was approximately 4.5 times higher than that of CSPM2.0. For the cells bearing the leukocyte common antigen, CD45^+^ (CD45-positive cells), a larger population exhibits CSPM0.1 retention at a longer time after injection. At 2 and 4 weeks after injection, the Cy7-positive populations in CSPM0.1 were 8.7 and 3.2%, respectively, indicating 3.9 and 8.5 times higher values than those corresponding to CSPM2.0. The population changes of Cy7-positive cells among the structural cells, such as epithelial (CD45^−^CD326^+^) and endothelial (CD45^−^CD31^+^) cells showed similar trends for both CSPM0.1 and CSPM2.0 injections, with overall higher values in the case of CSPM0.1. Figure 4h shows the confocal microscopy images of single cells isolated from the lungs, and the internalization of the injected particles in the cells can be observed. It is assumed that the smaller the particle size, the more actively they penetrate the organs through cellular internalization or cell junctions, increasing the possibility of interaction with leukocytes and facilitating movement to other organs through vascular circulation.

## Conclusions

In this study, we designed and synthesized fluorescent silica-based particles with different size distributions as a model of fine PM. The Cy7-doped silica PM was employed to perform the whole-body biodistribution imaging, ex vivo organ and tissue imaging, and flow cytometric analysis of single cells isolated from the lungs. The accumulation of small-sized PM in the lung decreased rapidly compared to larger particles, but had a longer residence time and was likely to be internalized into the immune system. In addition, migration to other organs was observed within a short period of inhalation, as opposed to relatively large particles. The population of PM accumulating in the lung cells was affected by the particle size distribution, and the size-dependent biodistribution patterns became more evident over time after injection.

This study could provide with insights on association between the accumulation of ultrafine particles in multiple organs, including the lungs, and adverse effects on the underling diseases of the organs. Although this study is limited to particle size dependence, if the comparison of results on diverse variables such as particle shape and surface state continues, it will be possible to build a biodistribution library for various model PM particles, which can be utilized in predicting PM toxicity.

## Materials and methods

### Materials

Cyanine 7 N-hydroxysuccinimide ester (Cy7-NHS) was purchased from Lumiprobe (FL, USA) and tetraethyl orthosilicate (TEOS, 98%) and 3-aminopropyltriethoxysilane (APTES) were purchased from Sigma-Aldrich (MO, USA). Aqueous ammonia (28–30%) was obtained from Samchun Chemicals (Pyeongtaek, Korea). Collagenase A and DNase I were purchased from Sigma-Aldrich, and ammonium-chloride-potassium (ACK) lysing buffer was purchased from Thermo Fisher Scientific (MA, USA). All antibodies were purchased as fluorochrome conjugates (Additional file 1: Table S1). A 70-μm cell strainer was procured from Falcon (NY, USA).

### Synthesis of Cy7-doped silica particulate matters

Cy7-doped silica particulate matters (CSPMs) were synthesized according to a previously reported method with slight modifications [35]. A Cy7-APTES conjugate was synthesized by stirring a mixed solution of Cy7-NHS and APTES in ethanol overnight at room temperature [36]. CSPM with a diameter of less than 100 nm (CSPM0.1) was synthesized using the following method. TEOS (2 mL), Cy7-APTES (2.64 mL) and ethanol (0.36 mL) were added to a 50 mL 1-neck round bottomed flask. A premixed solution (25 mL) containing ammonium hydroxide (1.5 mL) and ethanol (23.5 mL) was added to the above reaction mixture and stirred overnight at room temperature. CSPM0.1 was isolated and purified by centrifugation three times, followed by dispersion and storage in ethanol at 4 °C until further use. For the synthesis of CSPM with a diameter of approximately 2 μm (CSPM2.0), the same amounts of TEOS, Cy7-APTES, and ammonium hydroxide were used, but their concentrations were increased ~ 4.3 times. The synthesized particles were isolated by removing the ethanol and then dispersed in saline at a specific concentration prior to use.

### Characterization of CSPMs

Morphological analyses of CSPM0.1 and CSPM2.0 were performed using field-emission scanning electron microscopy (FE-SEM, Merlin Compact, Carl Zeiss, Jena, Germany). The particle size distribution and surface zeta potential were analyzed using a Zetasizer (Nano ZS, Malvern Instruments Ltd, Malvern, UK). The fluorescence emission spectra were recorded using a fluorescence spectrophotometer (FS2, Scinco, Seoul, Korea).

### Fluorescence stability studies and dye release profiling of CSPMs

To evaluate the fluorescence stability, the CSPMs, Cy7-APTES, and Cy7-NHS dispersed in saline were placed in a shaking incubator (Gaon Science, Seoul, Korea) at 37 °C in the dark. The fluorescence intensity change was analyzed by recording the fluorescence spectrum of each material at different time-points. To monitor the dye release behavior, the CSPMs dispersed in saline were placed in a shaking incubator at 37 °C, and after centrifugation for different durations, the fluorescence signal of the released dye remaining in the supernatant was analyzed.

### In vivo fluorescence imaging studies of CSPM-injected mice

All the experiments followed the guidelines of the Committee on Animal Research at the Korea Basic Science Institute (KBSI), and the protocol was approved by the local Institutional Review Committee on Animal Care (KBSI-IACUC-21-2). Male BALB/c nude mice (7–8 weeks old) were purchased from Nara Biotech (Seoul, Korea). All the mice were fed an alfalfa-free diet for at least one week before the start of the experiments. To evaluate the fluorescence intensity linearity and sensitivity toward in vivo image tracking, different concentrations of CSPMs (0.625, 1.25, 2.5, and 5 mg/mL) were prepared in saline and subcutaneously injected into the mouse abdomen. In vivo fluorescence signals were monitored using an in vivo imaging system (IVIS Spectrum; PerkinElmer, MA, USA) equipped with appropriate NIR filters (excitation = 675 nm, emission = 780 nm). For the biodistribution imaging studies, the saline CSPM solutions (3 mg/50 μL) were injected intratracheally into the mice under general anesthesia using ketamine and xylazine. At different time points (3 h, 2, 4, 8, 14, 21, and 28 days) after the injection, the mice were imaged and the organs (lung, heart, spleen, stomach, kidneys, liver, and intestine) were excised. After imaging the excised organs, flow cytometric and qualitative analyses were performed.

### Flow cytometric and qualitative analysis of the excised lungs

Flow cytometric analysis was performed on several cell types obtained from the excised lungs. The single cells from the lung tissue were obtained as described previously [33]. The excised lung was placed in 2 mL of RPMI 1640 medium (Gibco, NY, USA) containing 1 mg of collagenase A (Roche, Basel, Switzerland) and 0.4 mg of DNase I (Roche) and incubated at 37 °C for 45 min. Each lung tissue was chopped and the cells were filtered through a 70 μm cell strainer into a 50 mL conical tube using the plunger of a 3 mL syringe (Norm-Ject; Henke-Sass Wolf GmbH, Tuttlingen, Germany) and dispersed in 10 mL of RPMI medium. The cell pellets were obtained by centrifugation at 1500 rpm for 5 min, and the supernatant was discarded. The cells were dispersed in 5 mL of ACK lysing buffer and incubated at 37 °C for 5 min before adding 5 mL of RPMI. Thereafter, the cells were centrifuged, and the cell pellets were dispersed in 2 mL of PBS and passed through a 35 μm cell strainer into a FACS tube. The cells were centrifuged and resuspended in 1 mL of FACS buffer. Finally, the samples were divided and reacted with antibodies to perform flow cytometric analysis using a CytoFLEX flow cytometer (A00-1-1102; Beckman Coulter, CA, USA). Cellular microscopy images were acquired using a confocal laser scanning fluorescence microscope (Carl Zeiss, Oberkochen, Germany).

For elemental quantitative analysis of the Si obtained from the excised organs, the freeze-dried organs were analyzed using inductively coupled plasma-optical emission spectrometry (Optima 8300; Perkin Elmer, Waltham, USA).

### Fluorescence microscopic imaging of CSPMs in the lung tissues

For histological analysis of the excised lungs, the dissected lungs were embedded and frozen in Tissue-Tek optical cutting temperature compound (Sakura, Tokyo, Japan). Tissue blocks were sectioned at a thickness of 7 µm and stained with DAPI for nuclear staining. The frozen sections were scanned using a slide scanner system (Axio Scan.Z1; Carl Zeiss).

## Supplementary Information

**Additional file 1: Fig. S1.** (**a**) Cy7 encapsulation efficiency, loading amount of Cy7 in CSPM, and fluorescence intensity of CSPM with various amounts of Cy7 used for particle formulation. (**b**) Fluorescence intensity of CSPM with various amounts of Cy7 used for particle formulation. (**c**) Fluorescence intensity of 0.2 mg CSPM with various loading amounts of Cy7 (nmol). (**d**) FE-SEM images of the synthesized CSPM with various amounts of Cy7 used for particle formulation. Fluorescence signals were recorded using a microplate reader (VICTOR X2 Multilabel PerkinElmer, MA, USA; λ_ex_/λ_em_ = 660/750 nm). **Fig. S2.** The morphological stability of CSPMs. Representative FE-SEM images of (**a**) CSPM0.1 and (**b**) CSPM2.0 before (0 day) and after 11 and 21 days incubation in saline and 10% FBS contained media at 37 °C. **Fig. S3.** Fluorescence intensity changes of (**a**) CSPM0.1 (2.4 mg/ml) and (**b**) Cy7 dye (0.625 μg/ml) after incubating with various reactive oxygen species (2 μM H_2_O_2_, 10 mM GSH, 10 mM H_2_S) at different time points. Fluorescence signals were recorded using a microplate reader (VICTOR X2 Multilabel PerkinElmer, MA, USA; λ_ex_/λ_em_ = 660/750 nm). **Fig. S4. **Synthesis and characterization of CSPM2.0. (**a**) A representative FE-SEM image (scale bar = 1 μm). The inset shows HR-TEM image (scale bar = 2 μm). (**b**) Size distribution analysis of the synthesized CSPM2.0. (**c**) Surface zeta potentials of silica particulate matter (SPM2.0) without dye-doping and CSPM2.0. (**d**) Fluorescence spectra (excitation and emission scan modes) of CSPM2.0. **Fig. S5.** (**a**) FT-IR spectra of SPM0.1, CSPM0.1, SPM2.0, and CSPM2.0. (**b**) Magnified spectra of SPM0.1 and CSPM0.1. The bands at 795 and 445 cm^-1^ are assigned to Si–O–Si stretching and Si–O–Si bending, respectively. The absorption band at 1,053 cm^-1^ is assigned to the siloxane vibrations of (SiO)_n_ groups. The peaks at 3,268 and 1,634 cm^-1^ are attributed to O–H stretching band of the surface of silanol groups and residual water molecules. After doping of Cy7-APTES in silica particles, the new bands (1,524 cm^−1^ and 3,615 cm^−1^) are assigned to N–H bending and N-H stretching vibration of amine groups, respectively [1]. **Fig. S6.** The quantitative elemental analysis using ICP-OES for Si from the excised organs (n > 5) after injection of CSPM0.1 into the mice. The data shown are averages of >3 independent experiments. **Fig. S7. **Cell viability of RAW264.7 (mouse macrophage cell) and MRC-5 (human fibroblast cell) treated with CSPM0.1 and CSPM2.0 as a function of CSPM concentration. The cells were treated with CSPMs for 24 and 48 h and added the CellTiter 96 solution (Promega, WI, USA) and incubated for 1 h. Absorbance was measured at 490 nm using microplate absorbance spectrophotometer (BioRAD, CA, USA). The data shown are averages of three independent experiments. **Table S1.** Fluorochrome-conjugated antibodies and flow cytometer setup.

## Data Availability

The datasets in the current study are included in the published article or available from the corresponding author on reasonable request.

## Abbreviations

ACK: Ammonium-chloride-potassium
APTES: 3-Aminopropyltriethoxysilane
CSPM: Cy7-doped silica particulate matter
CSPM0.1: Cy7-doped silica particulate matter with a diameter of less than 0.1 μm
CSPM2.0: Cy7-doped silica particulate matter with a diameter of approximately 2.0 μm
Cy7-NHS: Cyanine 7 N-hydroxysuccinimide ester
FE-SEM: Field-emission scanning electron microscopy
NIR: Near-infrared
PM: Particulate matter
PM_0.1_: Particulate matter with a diameter of less than 0.1 μm
PM_10_: Particulate matter with a diameter of less than 10 μm
PM_2.5_: Particulate matter with a diameter of less than 2.5 μm
SNR: Signal-to-noise ratio
TEOS: Tetraethyl orthosilicate
