# Supplementary material for: Monitoring in vivo behavior of size-dependent fluorescent particles as a model fine dust
Source: J Nanobiotechnology. 2022 May 12;20:227. doi: 10.1186/s12951-022-01419-4 (PMC9097390; doi:10.1186/s12951-022-01419-4)
Supplement: Supplementary file 1 — Additional file 1: Fig. S1. (a) Cy7 encapsulation efficiency, loading amount of Cy7 in CSPM, and fluorescence intensity of CSPM with various amounts of Cy7 used for particle formulation. (b) Fluorescence intensity of CSPM with various amounts of Cy7 used for particle formulation. (c) Fluorescence intensity of 0.2 mg CSPM with various loading amounts of Cy7 (nmol). (d) FE-SEM images of the synthesized CSPM with various amounts of Cy7 used for particle formulation. Fluorescence signals were recorded using a microplate reader (VICTOR X2 Multilabel PerkinElmer, MA, USA; λex/λem = 660/750 nm). Fig. S2. The morphological stability of CSPMs. Representative FE-SEM images of (a) CSPM0.1 and (b) CSPM2.0 before (0 day) and after 11 and 21 days incubation in saline and 10% FBS contained media at 37 °C. Fig. S3. Fluorescence intensity changes of (a) CSPM0.1 (2.4 mg/ml) and (b) Cy7 dye (0.625 μg/ml) after incubating with various reactive oxygen species (2 μM H2O2, 10 mM GSH, 10 mM H2S) at different time points. Fluorescence signals were recorded using a microplate reader (VICTOR X2 Multilabel PerkinElmer, MA, USA; λex/λem = 660/750 nm). Fig. S4. Synthesis and characterization of CSPM2.0. (a) A representative FE-SEM image (scale bar = 1 μm). The inset shows HR-TEM image (scale bar = 2 μm). (b) Size distribution analysis of the synthesized CSPM2.0. (c) Surface zeta potentials of silica particulate matter (SPM2.0) without dye-doping and CSPM2.0. (d) Fluorescence spectra (excitation and emission scan modes) of CSPM2.0. Fig. S5. (a) FT-IR spectra of SPM0.1, CSPM0.1, SPM2.0, and CSPM2.0. (b) Magnified spectra of SPM0.1 and CSPM0.1. The bands at 795 and 445 cm-1 are assigned to Si–O–Si stretching and Si–O–Si bending, respectively. The absorption band at 1,053 cm-1 is assigned to the siloxane vibrations of (SiO)n groups. The peaks at 3,268 and 1,634 cm-1 are attributed to O–H stretching band of the surface of silanol groups and residual water molecules. After doping of Cy7-APTES in silic [file 12951_2022_1419_MOESM1_ESM.docx]

**Additional file 1**

**Monitoring in vivo behavior of size-dependent fluorescent particles as a model fine dust**

Taewoong Son^a,c,^^[[1]](#footnote-1)^, Youn-Joo Cho^a,c,1^, Hyunseung Lee^a^, Mi Young Cho^a,2^, Byeongwoo Goh^a^, Hyun Min Kim^a^, Phan Thi Ngoc Hoa^a,c^, Sun-Hee Cho^a^, Young-Jun Park^b^, Hye Sun Park^a,*^, and Kwan Soo Hong^a,c,*^

^a^ Research Center for Bioconvergence Analysis, Korea Basic Science Institute, Cheongju 28119, Korea

^b^ Environmental Disease Research Center, Korea Research Institute of Bioscience and Biotechnology, Daejeon 34141, Korea

^c^ Graduate School of Analytical Science and Technology, Chungnam National University, Daejeon 34134, Korea

KEYWORDS: air pollution, particulate matter, silica particle, biodistribution, *in vivo* image-tracking

**Fig. S1** (**a**) Cy7 encapsulation efficiency, loading amount of Cy7 in CSPM, and fluorescence intensity of CSPM with various amounts of Cy7 used for particle formulation. (**b**) Fluorescence intensity of CSPM with various amounts of Cy7 used for particle formulation. (**c**) Fluorescence intensity of 0.2 mg CSPM with various loading amounts of Cy7 (nmol). (**d**) FE-SEM images of the synthesized CSPM with various amounts of Cy7 used for particle formulation. Fluorescence signals were recorded using a microplate reader (VICTOR X2 Multilabel PerkinElmer, MA, USA; λ_ex_/λ_em_ = 660/750 nm).

**Fig. S2** The morphological stability of CSPMs. Representative FE-SEM images of (**a**) CSPM0.1 and (**b**) CSPM2.0 before (0 day) and after 11 and 21 days incubation in saline and 10% FBS contained media at 37 ̊C.

**Fig. S3** Fluorescence intensity changes of (**a**) CSPM0.1 (2.4 mg/ml) and (**b**) Cy7 dye (0.625 μg/ml) after incubating with various reactive oxygen species (2 μM H_2_O_2_, 10 mM GSH, 10 mM H_2_S) at different time points. Fluorescence signals were recorded using a microplate reader (VICTOR X2 Multilabel PerkinElmer, MA, USA; λ_ex_/λ_em_ = 660/750 nm).

**
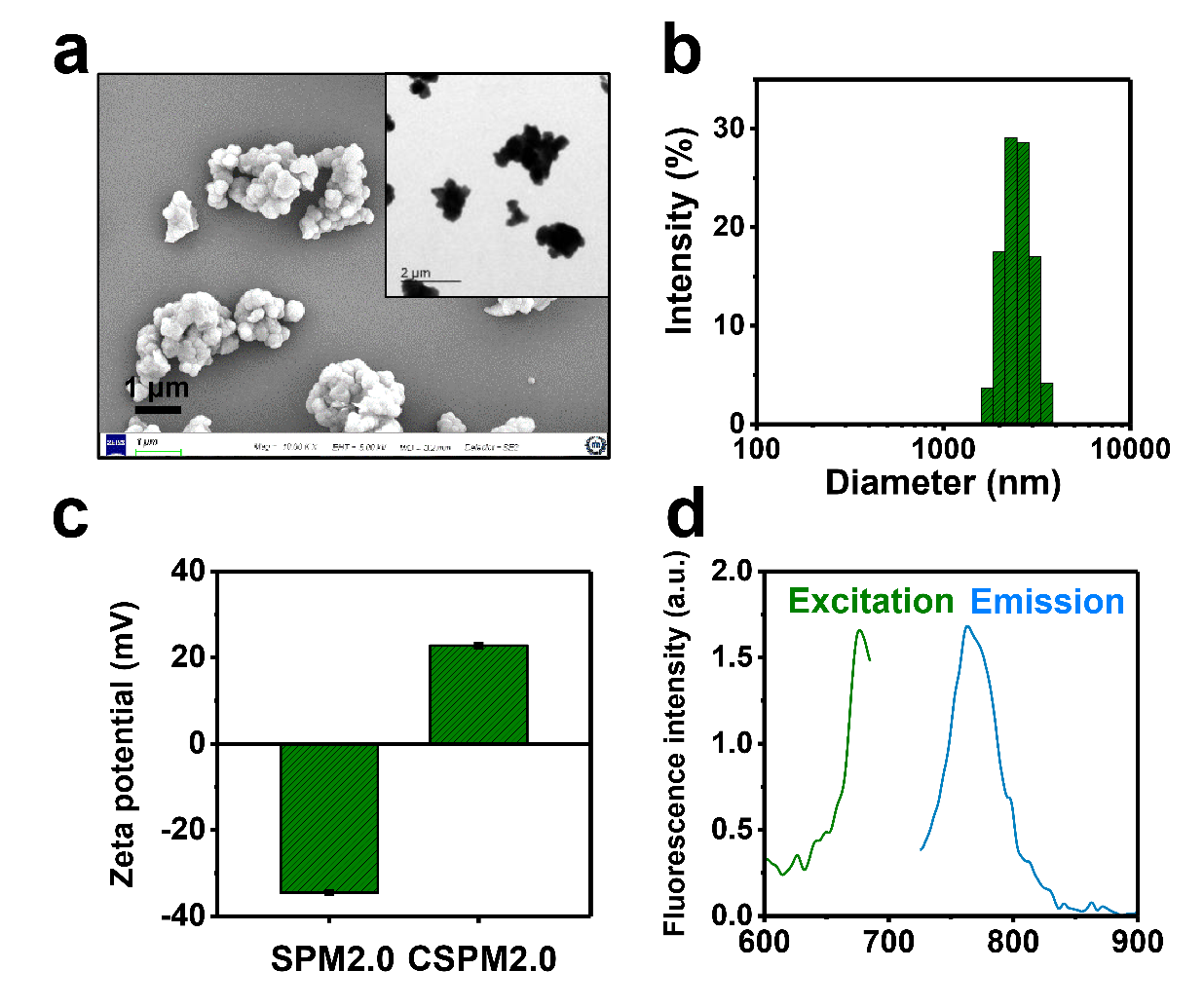
**

**Fig. S4** Synthesis and characterization of CSPM2.0. (**a**) A representative FE-SEM image (scale bar = 1 μm). The inset shows HR-TEM image (scale bar = 2 μm). (**b**) Size distribution analysis of the synthesized CSPM2.0. (**c**) Surface zeta potentials of silica particulate matter (SPM2.0) without dye-doping and CSPM2.0. (**d**) Fluorescence spectra (excitation and emission scan modes) of CSPM2.0.

**Fig. S5** (**a**) FT-IR spectra of SPM0.1, CSPM0.1, SPM2.0, and CSPM2.0. (**b**) Magnified spectra of SPM0.1 and CSPM0.1. The bands at 795 and 445 cm^-1^ are assigned to Si–O–Si stretching and Si–O–Si bending, respectively. The absorption band at 1,053 cm^-1^ is assigned to the siloxane vibrations of (SiO)_n_ groups. The peaks at 3,268 and 1,634 cm^-1^ are attributed to O–H stretching band of the surface of silanol groups and residual water molecules. After doping of Cy7-APTES in silica particles, the new bands (1,524 cm^-1^ and 3,615 cm^-1^) are assigned to N–H bending and N-H stretching vibration of amine groups, respectively [1].

**Fig. S6** The quantitative elemental analysis using ICP-OES for Si from the excised organs (n > 5) after injection of CSPM0.1 into the mice. The data shown are averages of >3 independent experiments.

**Fig. S7** Cell viability of RAW264.7 (mouse macrophage cell) and MRC-5 (human fibroblast cell) treated with CSPM0.1 and CSPM2.0 as a function of CSPM concentration. The cells were treated with CSPMs for 24 and 48 h and added the CellTiter 96 solution (Promega, WI, USA) and incubated for 1 h. Absorbance was measured at 490 nm using microplate absorbance spectrophotometer (BioRAD, CA, USA). The data shown are averages of three independent experiments.

| **Marker** | **Clone** | **Fluorochrome** | **Company / #** | **Excitation laser** | **Emission filter** |
| --- | --- | --- | --- | --- | --- |
| CD45  (Leukocytes) | I3/2.3 | PE | BioLegend / 147712 | 488 | 585/42 |
| CD326 | G8.8 | Alexa488 | BioLegend / 118210 | 488 | 525/40 |
| CD31 | 390 | eFluor450 | Invitrogen / 48-0311-82 | 405 | 450/45 |
| CSPMs | - | Cy7 | - | 638 | 780/60 |

**Table. S1** Fluorochrome-conjugated antibodies and flow cytometer setup.

**Reference**

1. Azarshin S, Moghadasi J, A Aboosadi Z. Surface functionalization of silica nanoparticles to improve the performance of water flooding in oil wet reservoirs. Energy Exploration & Exploitation. 2017;35:685–97.

1. These authors equally contributed to this work.

   ^2^ Present address: SKKU Advanced Institute of Nanotechnology (SAINT), School of Chemical Engineering, Sungkyunkwan University, Suwon 16419, Korea.

   * Corresponding author. E-mail addresses: hspark@kbsi.re.kr (H. S. Park) and kshong@kbsi.re.kr (K. S. Hong). [↑](#footnote-ref-1)
